# Acute post-infectious cerebellar ataxia due to co-infection of human herpesvirus-6 and adenovirus mimicking myositis

**DOI:** 10.1186/s13052-014-0098-y

**Published:** 2014-11-26

**Authors:** Aldo Naselli, Giovanna Pala, Federico Cresta, Martina Finetti, Roberta Biancheri, Salvatore Renna

**Affiliations:** UOC First Aid and Emergency Department, Istituto Giannina Gaslini – Ospedale Pediatrico IRCCS, Largo G. Gaslini 5, 16147 Genova, Italy; Neuroscience Department, Istituto Giannina Gaslini – Ospedale Pediatrico IRCCS, Largo G. Gaslini 5, 16147 Genova, Italy

**Keywords:** Human herpes virus-6, Adenovirus, Hypotonia, Weakness, Ataxia

## Abstract

Acute cerebellar ataxia (ACA) is a relatively common neurological disease in children. Most common types of ACA are acute post-infectious (APCA) and acute disseminated encephalomyelitis (ADEM). Less common but important causes include opsoclonus-myoclonus syndrome (OMS) and acute cerebellitis. Cerebellar neoplasms and acute hydrocephalus are additional causes of paediatric ataxia. APCA is the most common cause of ACA in children, comprising about 30-50% of total cases. This is a report about an immunocompetent 4-yrs-old male affected by APCA, due to co-infection by human herpesvirus-6 (HHV-6) and adenovirus, with symptoms mimicking myositis.

## Background

Acute cerebellar ataxia (ACA) is a relatively common neurological disease in children.

Common causes are acute post-infectious ataxia (APCA), intoxications and acute disseminated encephalomyelitis (ADEM). Less common but important causes include opsoclonus-myoclonus syndrome (OMS) and acute cerebellitis. Cerebellar tumours and acute hydrocephalus are rare causes of paediatric ataxia.

APCA is the most common cause of acute ataxia in children, comprising about 30-50% of the total cases [1]. The most typical age is 2–5 yrs. Symptoms are typically characterized by the onset, in previously healthy patient, of gait alterations and/or inability to perform coordinated movements; these symptoms usually regress within 72 hours. The prognosis is good with a high rate of spontaneous resolution and no treatment is indicated.

Here we present the case of a patient with acute onset of weakness and muscle pain, evolving into cerebellar ataxia, due to adenovirus and human herpesvirus 6 (HHV-6) co-infection.

## Case report

A previously healthy 4-year-old boy came to our emergency department (ED) for the acute onset, during previous 12 hours, of lower limbs pain, associated with difficulties in maintaining upright posture up to complete refusal of walking. The pain was not modified by treatment with NSAID (oral ibuprofen, 15 mg/kg/dose). Parents reported a febrile upper airways infection approximately 15 days before, followed by skin rash spread. Trauma was denied. The patient looked ill, with normal vital signs, afebrile; general physical examination was negative. The first neurological examination showed muscle weakness and upper and lower extremities hypotonia, brisk tendon reflexes at limbs, either antigravity tests, autonomous sitting and position were impossible, normal speech pattern. The child refused walking and he was not able to attend his normal activities. The palpation of muscle bellies showed tenderness at the proximal lower limb and the passive mobilization of the knee and hip joints caused pain.

The patient was hospitalized to complete diagnostic investigations. Lab tests at admission were normal (complete blood count, C-reactive protein, CK, LDH, C3, C4, hepatic and renal function, urinary catecholamines). Research by polymerase chain reaction (PCR) on throat swab showed positivity for adenovirus-DNA. Abdominal and hip joints ultrasound as well as pelvis and lower limbs X-ray were negative. Lumbar puncture was performed on the day of admission: cerebrospinal fluid (CSF) analysis and culture resulted negative. Research for oligoclonal IgG bands by immunoblot appeared positive both in cerebrospinal fluid and in serum sample. Research by PCR for parvovirus B19, adenovirus, cytomegalovirus (CMV), Epstein-Barr virus (EBV), herpes simplex virus 1 and herpes simplex virus 2, HHV-6, *Mycoplasma pneumoniae*, varicella zoster virus (VZV) and enterovirus in CSF were negative. Serologic analysis on blood sample resulted positive for adenovirus and HHV6 – DNA (specific IgA).

Brain and spinal cord magnetic resonance imaging (MRI) with and without contrast, STIR Total Body MRI and electrophysiological investigations (motor and sensitive nerve conduction velocity studies, with F-wave and H-reflex) resulted to be negative.

Five days after admission the child was able to stand and walk with support and cerebellar ataxic gait was evident as well as fine tremors and finger-to-nose test frenage. A spontaneous and progressive recovery of motor activity up to a complete normalization occurred around 20 days after. On the base of performed investigations and clinical evolution, it was possible to make a diagnosis of APCA.

## Conclusions

We present a pediatric case of APCA due to co-infection of HHV-6 and adenovirus with acute onset of weakness and muscle pain at the first visit in ED. APCA is an immune-mediated neurologic disease, relatively common in children, due to an inflammatory cerebellar process. Generally, gait and truncal ataxia with dysmetria are common presenting features. APCA is usually the result of a post-infectious inflammation: this autoimmune phenomenon could be triggered by an inciting infection (or immunization), with the subsequent production of autoantibodies which cross-react with cerebellar epitopes [2,3]. Children with APCA are normally alert and interactive and a recent illness is commonly reported (in almost 70% of the cases); VZV is the most common pathogen reported in literature, occurring in 26% of cases [4-6]; also infections by coxsackie virus, echovirus, EBV, pertussis, rotavirus, measles, Influenza A and B, *Mycoplasma*, Parvovirus B19 [7,8] and, as in our patient, HHV-6 and Adenovirus are reported as possible infectious triggers of APCA. A quarter of the cases, in which it is not possible to identify a cause, is therefore classified as idiopathic [4]. CSF examination is usually negative, but in some cases oligoclonal gamma-globulin bands or lymphocytic inflammatory reaction (as mild pleocytosis is found in 25% of cases) can be detected; microbiological researches on CSF rarely detect direct viral infection of CNS. Brain imaging (MRI, CT scan) is usually negative [1,4].

The most severe manifestation of this pathological spectrum is represented by cerebellitis, characterized by acute neurological and systemic symptoms associated with neuroimaging cerebellar abnormalities [9,10] and meningeal involvement [11]. The differential diagnosis of acute forms of cerebellar ataxia in children is quite wide; a complete medical history and physical examination can easily suggest a correct diagnosis, although some symptoms, such as major pain, should suggest further tests.

Our study reports for the first time a myositis-like onset (muscle pain and inability of walking), in a patient who later developed a cerebellar ataxia. This case suggests that ACA should be considered in children who had primary muscle pain and weakness and in a second time ataxia and/or other acute neurological symptoms.

We believe that this manuscript could be useful for the management of patients with muscle pain and hypotonia with previous history of respiratory tract infection. APCA should be always suspected, particularly in presence of cerebellar symptoms.

## Consent

Written informed consent was obtained from the patient guardian for publication of this Case Report and any accompanying images. A copy of written consent is available for review by the Editor-in-Chief of this journal.

## Abbreviations

ACA: Acute cerebellar ataxia
APCA: Acute post-infectious ataxia
ADEM: Acute disseminated encephalomyelitis
OMS: Opsoclonus -myoclonus syndrome
HHV-6: Human herpesvirus 6
ED: Emergency department
PCR: Polymerase chain reaction
CSF: Cerebrospinal fluid
CMV: Cytomegalovirus
EBV: Epstein-Barr virus
VZV: Varicella zoster virus
MRI: Magnetic resonance imaging
CNS: Central neurologic system
